# The TrueBlue study: Is practice nurse-led collaborative care effective in the management of depression for patients with heart disease or diabetes?

**DOI:** 10.1186/1471-2296-10-46

**Published:** 2009-06-23

**Authors:** Mark Morgan, James Dunbar, Prasuna Reddy, Michael Coates, Robert Leahy

**Affiliations:** 1 Greater Green Triangle University Department of Rural Health, Flinders and Deakin Universities, Warrnambool, Victoria, Australia

## Abstract

**Background:**

In the presence of type 2 diabetes (T2DM) or coronary heart disease (CHD), depression is under diagnosed and under treated despite being associated with worse clinical outcomes. Our earlier pilot study demonstrated that it was feasible, acceptable and affordable for practice nurses to extend their role to include screening for and monitoring of depression alongside biological and lifestyle risk factors. The current study will compare the clinical outcomes of our model of practice nurse-led collaborative care with usual care for patients with depression and T2DM or CHD.

**Methods:**

This is a cluster-randomised intervention trial. Eighteen general practices from regional and metropolitan areas agreed to join this study, and were allocated randomly to an intervention or control group. We aim to recruit 50 patients with co-morbid depression and diabetes or heart disease from each of these practices. In the intervention group, practice nurses (PNs) will be trained for their enhanced roles in this nurse-led collaborative care study. Patients will be invited to attend a practice nurse consultation every 3 months prior to seeing their usual general practitioner. The PN will assess psychological, physiological and lifestyle parameters then work with the patient to set management goals. The outcome of this assessment will form the basis of a GP Management Plan document. In the control group, the patients will continue to receive their usual care for the first six months of the study before the PNs undergo the training and switch to the intervention protocol. The primary clinical outcome will be a reduction in the depression score. The study will also measure the impact on physiological measures, quality of life and on patient attitude to health care delivered by practice nurses.

**Conclusion:**

The strength of this programme is that it provides a sustainable model of chronic disease management with monitoring and self-management assistance for physiological, lifestyle and psychological risk factors for high-risk patients with co-morbid depression, diabetes or heart disease. The study will demonstrate whether nurse-led collaborative care achieves better outcomes than usual care.

## Background

Coronary heart disease (CHD) and type 2 diabetes mellitus (T2DM) are both major causes of disability. The incidence of T2DM is now reaching epidemic proportions in Australia [1] and overseas [2], particularly as the population ages and becomes more obese [3]. Within 20 years diabetes will become the leading contributor to the overall burden of disease in Australia [4] with Australian health care expenditure projected to increase to $7 billion by 2023 [5]. CHD already affects over 300,000 people in Australia and remains the most common cause of death [6]. Both diabetes and heart disease are associated with a number of serious complications, each with its own cost.

There is evidence that the presence of depression in patients with CHD or T2DM leads to increased morbidity and mortality [7,8], but co-morbid depression is often missed in routine consultations within general practices [9]. One difficulty is that the traditional model used by general practices is one in which visits to the general practitioner (GP) are initiated by patients when they feel that their needs warrant a consultation. Such visits are usually episodic and cease when the immediate symptoms are relieved. Consequently, such patients miss out on regular monitoring of their chronic disease so that risk factors go unrecognised [9].

A collaborative model is one in which the care delivery has multiple components that address these problems [10] and has been shown to have good results in treating depression [11]. It relies on a case manager to coordinate that care. Hickie and McGorry [12] summarise the planned episodes of care that include:

• Use of evidence based guidelines;

• Systematic screening and monitoring of risk factors;

• Timetabled recall visits;

• New or adjusted roles for team members;

• Information support for the clinician;

• Enhanced patient self-management;

• Identified case manager to coordinate care;

• A means of effective communication between all members of the care team; and

• Audit information for the practice.

The True Blue study described in this paper extends an exploratory trial to adapt the successful IMPACT model of collaborative care for depression in the USA [10] to an Australian primary health care setting. The case manager in this study is the practice nurse (PN). The model of practice nurse-led collaborative care was demonstrated in six general practices in rural southern Australia, with 332 patients recruited [13]. In this exploratory trial a practice nurse training programme was developed for chronic disease management, introducing depression screening and counselling techniques to assist with self-management. Electronically based multi-purpose tools were designed and tested to allow outcome data to be collected to inform coordinated medical care and patient self-management. The trial found depression in 34% of patients with CHD or T2DM, and demonstrated that the practice nurse-led, collaborative care model was both feasible and acceptable.

The role of practice nurses in collaborative care of chronic disease is being investigated both in Australia and overseas. [14-16]. The features of the True Blue study are that the programme

• routinely screens for depression;

• monitors depression severity over time for participating patients;

• uses the existing work force and funding arrangements to potentially make the model more widely applicable;

• uses consultations with practice nurses that allow collection of physiological measurements, monitoring of lifestyle and mental health risks and setting of patient goals; and

• is linked with appointments to the patient's usual general practitioner (GP).

The present study works with this subset of patients who have co-morbid depression and aims to demonstrate improved clinical outcomes for this higher-risk group through measurements of physiological, mental health and lifestyle parameters at regular intervals throughout the study period. It will further test and implement this collaborative care model that is focussed on the patient. An important aspect is that patients, in collaboration with the practice nurse, will develop up to three goals that they feel will be able to help reduce their risk factors, thus making patients active participants in their own health care.

The model is also intended to demonstrate how the care of co-morbid depression, heart disease and diabetes can be funded successfully using Australian Medicare Benefits Schedule (MBS) Item numbers. It will develop training programmes for PNs in screening, assessment and management of patients with co-morbid depression and heart disease or diabetes, and evaluate the feasibility of PNs to carry out screening and assessment.

### Objectives of this study

The primary objective of this study is to determine whether practice nurse-led collaborative care is better than the usual method of GP-led episodic care for the management of co-morbid depression in patients with heart disease or diabetes by testing whether there is an improvement in the depression score at the end of the study. The goal is to achieve a 50% reduction in that score. It will also test whether it is a practical way to manage this complex and increasing chronic-disease burden. It will introduce an hour-long consultation with the practice nurse in which patient goal setting forms an important proactive part of the patient care. One key strategy is that the patient outcomes will be reviewed every three months over an entire year and patient goals altered accordingly. Other objectives are to demonstrate that the model of care can use existing clinical staff and be funded within current Medicare arrangements, and that it can be used in large and small practices across rural and urban settings.

## Methods/design

**Figure 1:**
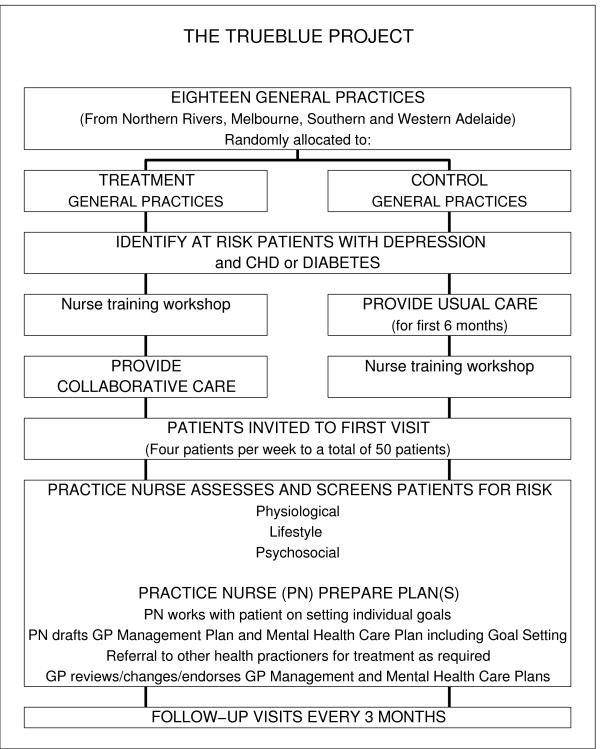
**Flowchart of the TrueBlue project**.

This study is a cluster-randomised intervention trial in which general practices were allocated either to an intervention group in which nurse-led collaborative care is to be undertaken or to the control group in which usual GP-led care is to be continued. Ethics approval for the project was obtained from Flinders University Social and Behavioural Research Ethics Committee, approval number 4164. Figure 1 shows the flowchart of the TrueBlue study.

### Survey forms

#### Patient Health Questionnaire (PHQ-9)

The PHQ-9 questionnaire will be used to measure and monitor depression over time. It is self-administered, suitable for face-to-face and postal responses and for research. Its brevity and reliability as a valid measure of depression severity make it a useful clinical and research tool [17]. The form has nine questions that are simply scored from 0 (no problems) to 3 (problems nearly every day). Each of these assesses various physiological and mood indicators of depression, and are combined to form the total PHQ-9 score. Scores 5–9 indicate mild depression, 10–14 moderate depression, 15–19 moderately severe, and scores above 19 indicate severe depression.

#### SF36 (Version 2)

Health and lifestyle will be measured using version 2 of the SF-36 questionnaire. This questionnaire is a multi-purpose, short-form health survey that provides a profile of health and well-being as well as a psychometrically-based physical and mental health summary measures and a preference-based health utility index [18]. This questionnaire provides a suite of generic measures rather than specific ones, but has proven to be useful in surveys of general and specific populations in differentiating the health benefits produced by a wide range of different treatments.

#### The GP Management Plan (GPMP)

Critical to collaborative care in chronic-disease management is a system that ensures the coordination of care between the GP and other health-care providers and assists patient self-management. This document, the GP management plan (GPMP), was developed by the GGT UDRH in collaboration with each of the practices in the programme. The design of this multi-purpose document enables it to serve the following functions, within the limits of current electronic medical-record applications, with minimum data entry:

• Provide a single template covering T2DM and CHD diagnoses;

• List patient medical history;

• List medication used, including prescribed medication, aspirin and antidepressant use;

• Record physiological risk factors, including blood pressure, weight, height, body-mass index (BMI), waist circumference, microalbuminuria, lipid profile and, for diabetic patients, HbA1c;

• Record lifestyle risks, including smoking, alcohol consumption and exercise regime;

• Record the psychosocial risks and PHQ-9 depression scores, including referrals to and consultations with a mental-health worker;

• Checklist of preventative activities and targets recommended in National Heart Foundation and Diabetes Australia guidelines;

• Individual patient goals, targets and agenda for future consultations;

• List referrals to specialists, allied health and community resources;

• Timetabled recall;

• Help practices complete the requirements to claim Medicare rebates for care planning and diabetes cycle of care; and

• Automatically generate de-identified data for research and audit.

The GPMP includes an office-use summary table containing only data that are relevant to the research. These data are identified only by a unique patient ID number assigned by the patient's practice. No personal details are forwarded to the research team. The patient information in the GPMP office-use summary is listed in the Appendix.

### Sample size

Nine intervention and nine control practices, each with 50 patients, will be required to detect a minimum 2-point reduction in the PHQ-9 score at the 0.05 significance level with 80% power. These patient and practice numbers were determined assuming a change in PHQ-9 with standard deviation of 5.1 as observed in the pilot study [13], a drop-out of no more than 33%, and an intra-cluster correlation of 0.04. Note that detecting a 2-point reduction in the overall PHQ-9 score is a more-stringent requirement than this project's objective of achieving a 50% reduction in that score.

### Practice recruitment

Practices from three regions (Adelaide, inner Melbourne and the NSW Northern Rivers area) were selected to undertake the study. Practices were selected from these regions using a range of criteria, including size and capacity, and assessable electronic medial records capable of generating an electronic registry of patients with CHD or T2DM. Practices also needed to have a PN available to lead the collaborative care.

Information about the study was circulated to these practices to explain the benefits of the True Blue project to their GPs, PNs and practice managers. These were followed up with orientation evenings that provided further detail. GPs who were interested in the project and who were able to guarantee protected time for their PN(s) to undertake the collaborative care were invited to join and their clinics included in the project, until six practices from each of the three regions had been selected.

#### Randomisation

Nine practices were randomly allocated to the intervention group in which nurse-led collaborative care will be undertaken. The remaining nine practices were allocated to the control group in which the usual GP-led care will continue for a period of six months. At the end of this period, the control practices will be invited to implement the practice nurse-led model of collaborative care and to send practice nurses to a training workshop (see below).

### Patient recruitment

Within each general practice, a mailing list of patients with a diagnosis of CHD or T2DM is generated. Patients who are either under 18 years of age or in residential care are excluded from the study. Eligible patients are added to the master list as potential participants in the programme and assigned a unique identification (ID) number. All documentation or data forwarded to the researchers for the patient is identified only by this unique ID number. Personal details of patients, including names and addresses, are retained by the clinic concerned.

An information package is posted from the practice to each of these patients. It includes a standardised personal letter from their GP explaining the study, information brochures, a PHQ-9 questionnaire, and a consent form indicating the patient's willingness to participate in the programme. Each patient is asked to complete and return the PHQ-9 and consent forms. The returned PHQ-9 questionnaire from consenting patients is examined to identify PHQ-9 scores above 5, indicating presence of at least mild depression. These patients are invited to join the study. Patients who do not respond to the first mail-out are sent a reminder letter approximately two weeks later inviting them to participate, with further reminder letters, telephone calls and personal invitations when visiting clinics, until 50 patients have been recruited.

### Inclusion and exclusion criteria

Practices with an electronic-medial-records system capable of generating an electronic registry of patients with CHD or T2DM and with a PN available to lead the collaborative care are eligible to participate. Patients with either T2DM or CHD are eligible to participate, provided that they were over 18 years of age and are not in residential care.

### Practice nurse training workshop

A two-day training workshop was organised for the PNs from the intervention practices to prepare them for enhanced roles in nurse-led collaborative care. This workshop introduced the rationale of the collaborative care model before presenting a range of topics to prepare PNs for their additional roles. Topics presented in the workshop include:

• Screening for depression;

• Identification and measurement of physiological risk factors such as high cholesterol, blood pressure, blood glucose and central obesity;

• Lifestyle risk factors such as smoking, poor nutrition, alcohol and physical inactivity;

• Training to educate patients in diabetes and heart disease risk reduction;

• Training in assisting patients with goal setting and problem solving;

• Coordinating referrals and timetabled follow up; and

• Preparing the draft GP Management Plan.

### Intervention programme

Each patient's session with the PN is scheduled to take approximately one hour. During their initial (baseline) session, each patient completes the SF36v2 questionnaire and a new PHQ-9 questionnaire if the earlier one is more than two weeks old. Their current medication list is also updated to include over-the-counter products. The PN assesses the patient, beginning by measuring their physiological parameters, reviews the potential risk factors (biological, lifestyle, psychological) and develops up to three lifestyle goals with the patient that he/she feels are achievable in reducing his/her risk factors. The SNAP (Smoking, Nutrition, Alcohol, Physical exercise) assessment , for example, can be a useful guide. The PN and patient identify possible barriers to achieving these goals and discuss enabling methods that may overcome these barriers. The PN may also suggest other professional services that may assist the patient in improving his/her outcomes, such as a dietician or counsellor. The PN may also supply educational material to assist patients in understanding their condition and meeting their goals. The PN completes the consultation by setting the review appointment date, and identifying any future tests that may be required beforehand. Patients responding positively to the ninth question on the PHQ-9, which assesses suicidal ideation, are immediately referred to their GP. The practice nurse consultation is scheduled to take about 45 minutes to complete. The remaining 15 minutes is set aside to add these data to the GP management plan (GPMP) before this is forwarded to the GP. The draft GPMP document becomes a readily accessible information support document for medication changes and referrals. The GP completes the plan during the consultation with the patient, providing the patient with a copy of the completed GPMP document.

An important aspect of this collaborative care programme is that patients are recalled systematically to monitor the progress of their care. Another important aim is that the nurse-led care can be completely self-funded using the normal Medicare item numbers. Consequently, a thirteen-week interval was chosen as the recall period. The recall visits are booked at the time of the previous visit. During each review, patients will complete a new PHQ-9 questionnaire so that any changes to their mental health can be monitored. Additional therapies or new strategies can be considered during the consultations if the PHQ-9 score has not improved by at least 50% or dropped below 5. These strategies may include changing or adding medication or referral to a mental health professional. The PN and patient then re-evaluate the goals from the previous consultation. In the second (6-month) and fourth (12-month) consultations, the patient will complete a new SF36v2 form to assess changes in quality of life.

### Data collection, verification and analysis

The research team includes a practice facilitator in each region to offer support to practice nurses and to monitor progress of the research programme. Monthly teleconference meetings are held to identify common issues and problems so that these can be addressed in a timely manner.

Data collection begins in the practice with the PNs entering patient data into their respective practice electronic databases, and then using these data to generate the GPMP for each patient. The GPMP includes a de-referenced, office-use-only summary table, shown in the Appendix, with the data that are relevant to the research. The PN copies the de-identified summary table from the practice database into a data-validation spreadsheet supplied by the GGT UDRH. The spreadsheet checks the GPMP summary table for completeness, consistency and accuracy. It highlights missing data and data that lie outside expected ranges that may be a result of typographical errors. These provide a warning to allow the PNs to recheck the data. Once verified, the PN forwards the validated GPMP summary data to the research team at GGT UDRH, where a final check is performed before their data are added to the master database. 

Independent groups *t*-tests and *χ*^2 ^tests will be used with baseline measures to check intervention and control for any imbalance. Clinical measures and changes (between baseline and follow-up) in outcomes of participants will be compared with measurements of controls. The difference in the change in the continuous outcome measures (particularly the PHQ-9 score) between the two studied groups will be analysed using linear mixed models, treating "group" as a between-subject factor and "time" as a within-subject factor. Categorical outcome variables will be analysed using generalised estimating equations (GEE), following the same approach. The intention-to-treat principle will be adhered to, and sensitivity analysis will also be carried out. Regression analysis, including logistic regression, will be used to identify baseline variables that predict successful outcomes and adherence.

### Acceptability interviews

On completion of the programme, a series of structured interviews with PNs and GPs will be undertaken to discuss the nurse-led collaborative care model and its perceived strengths, weaknesses and barriers. We will examine how the role of the PN is changed, concentrating on confidence and expertise in leading the new model of care. We will examine how the GPs have or have not accepted the model of care and determine any problems and issues that may have arisen in implementing the model.

We will also undertake interviews with a randomly selected list of patients. These interviews will be conducted by telephone to preserve patient anonymity. We will discuss the perceived strengths and weaknesses of the collaborative care model from the patient's point of view, and examine barriers and enablers to the model.

## Discussion

The strength of this programme is that it provides a full package of chronic disease management techniques, based on Wagner's chronic disease management (CDM) model [19]. The programme involves reading the practice electronic medical record to generate a master list of patients who satisfy the prescribed selection criteria. These patients are invited to begin the collaborative care process, and are systematically recalled at the prescribed review period. The collaborative care process is audited using patient feedback. Medicare funding will mean practices are remunerated for the more intense patient intervention.

The programme uses the existing workforce but involves an enhanced role for the practice nurses and so is applicable for wider roll out in using this potentially under-utilised resource. The practice nurses gain enhanced skills in the CDM set up and management that will be a useful model for patients with other chronic diseases.

A full range of outcome measures is reported and added to the electronic database. Consequently, practices will know what happens to patients (process of care) and will be able to identify changes in classic risk factors, depression symptoms and affordability within the Australian health system.

We note that practices need to be large enough to have available a practice nurse to participate in the programme. This study is only funded for 15 months of data collection but early feedback from participating practices indicates they are likely to choose to continue this nurse-led collaborative model of care beyond the lifetime of the research.

## Competing interests

The authors declare that they have no competing interests.

## Authors' contributions

MM designed and will implement the project. JD and PR conceived the research idea and provide strategic direction. MC manages data collection and the master database. BL manages the project, overseeing the model at each practice. All authors have read and approved the final manuscript.

## Appendix

The patient information provided in the GP Management Plan office-use summary section is shown below.

Patient's True Blue Record Number

GPMP or REVIEW

Diagnosis (CHD/T2DM/Both)

Date of birth

Gender

Ethnicity (ATSI) status

Patient speaks language other than English at home

Date of this service

Date of previous GP Management Plan (if prepared)

Date of last GP Mental Health Care Plan (if done)

SF36v2 completed

Smoking

Alcohol consumption

Current weight

Height

BMI

Current BP

Current waist circumference

Patient exercises 30 minutes per day, 5 days per week?

Total Cholesterol

Triglycerides

LDL

HDL

Previous episode of depression or anxiety?

Type of current treatment

Currently taking antidepressant medication?

Referred to Mental Health Worker?

Currently seeing Mental Health Worker?

Has the patient been referred to an exercise program?

Is the patient attending an exercise program?

PHQ9 total score

PHQ9 difficulty score

Proposed review date

Aspirin use

Goal 1 for the next three months

Goal 2 for the next three months

Goal 3 for the next three months

Previous goal 1 (Met/Partially met/Re-negotiated)

Previous goal 2 (Met/Partially met/Re-negotiated)

Previous goal 3 (Met/Partially met/Re-negotiated)

T2DM only

HbA1c

Micoalbuminuria results (normal/raised)

Month of last professional eye exam

Date of last recorded foot check

## Pre-publication history

The pre-publication history for this paper can be accessed here:


